# The Vasopressin Receptor 2 Mutant R137L Linked to the Nephrogenic Syndrome of Inappropriate Antidiuresis (NSIAD) Signals through an Alternative Pathway that Increases AQP2 Membrane Targeting Independently of S256 Phosphorylation

**DOI:** 10.3390/cells9061354

**Published:** 2020-05-29

**Authors:** Marianna Ranieri, Maria Venneri, Tommaso Pellegrino, Mariangela Centrone, Annarita Di Mise, Susanna Cotecchia, Grazia Tamma, Giovanna Valenti

**Affiliations:** 1 Department of Biosciences, Biotechnologies and Biopharmaceutics, University of Bari, 70125 Bari, Italy; marianna.ranieri@uniba.it (M.R.); maria.venneri@uniba.it (M.V.); tommaso.pellegrino89@hotmail.com (T.P.); mariangela.centrone@uniba.it (M.C.); annarita.dimise@uniba.it (A.D.M.); susanna.cotecchia@uniba.it (S.C.); grazia.tamma@uniba.it (G.T.); 2 Istituto Nazionale di Biostrutture e Biosistemi, 00136 Roma, Italy; 3 Center of Excellence in Comparative Genomics (CEGBA), University of Bari, 70125 Bari, Italy

**Keywords:** vasopressin, V2R, NSIAD, AQP2, Rho kinase (ROCK)

## Abstract

NSIAD is a rare X-linked condition, caused by activating mutations in the *AVPR2* gene coding for the vasopressin V2 receptor (V2R) associated with hyponatremia, despite undetectable plasma vasopressin levels. We have recently provided in vitro evidence that, compared to V2R-wt, expression of activating V2R mutations R137L, R137C and F229V cause a constitutive redistribution of the AQP2 water channel to the plasma membrane, higher basal water permeability and significantly higher basal levels of p256-AQP2 in the F229V mutant but not in R137L or R137C. In this study, V2R mutations were expressed in collecting duct principal cells and the associated signalling was dissected. V2R-R137L and R137C mutants had significantly higher basal pT269-AQP2 levels -independently of S256 and PKA-which were reduced to control by treatment with Rho kinase (ROCK) inhibitor. Interestingly, ROCK activity was found significantly higher in V2R-R137L along with activation of the Gα12/13–Rho–ROCK pathway. Of note, inhibition of ROCK reduced the basal elevated osmotic water permeability to control. To conclude, our data demonstrate for the first time that the gain-of-function mutation of the V2R, R137L causing NSIAD, signals through an alternative PKA-independent pathway that increases AQP2 membrane targeting through ROCK-induced phosphorylation at S/T269 independently of S256 of AQP2.

## 1. Introduction

NSIAD is a rare disorder characterized by impaired renal capacity to excrete a free water load and hyponatremia associated with undetectable plasma vasopressin levels due to a gain-of-function mutation in the vasopressin receptor type 2 (V2R). The first description of the disease was published in 2005 by Feldman and co-workers who discovered that two unrelated male infants having severe hyponatremia and undetectable vasopressin levels, carried an R137C or R137L mutation in the V2R gene [1]. Since its discovery, however, only a few cases have been described (about 30) and among them, R137C was found to be the most frequent mutation [2,3].

Arginine 137 is highly conserved in V2R and is located in the DRY/H domain of the second intracellular loop, a key motif for several aspects of GPCR functions [4]). Several studies indicate that this site may be important for the stabilization of the receptor in its active or inactive form [5]. Interestingly, the substitution of this residue from arginine to histidine causes an opposite disease Nephrogenic Diabetes Insipidus (NDI), characterized by the kidney inability to concentrate urine which results in polyuria and polydipsia.

It has been shown that both R137H and R137L/C mutants have a high rate of arrestin-dependent constitutive internalization, [6,7,8,9]. In contrast, three other more recently discovered gain-of-function mutations causing NSIAD, F229V, I130N and L312S, displayed constitutive cAMP generation without constitutive beta-arrestin recruitment and are sensitive to the inverse agonist tolvaptan [2,3,10]. Another interesting study identified two families with NSIAD phenotype carrying a mutation in the G protein a-subunit coupled to the receptor [11]. Therefore, mutations causing NSIAD appear to be associated with the activation of several different intracellular pathways.

Besides water restriction and urea supplementation (as osmotic agent), a logical therapeutic approach for NSIAD patients would be the use of V2R inverse agonists. However, in vitro studies have shown that tolvaptan and satavaptan reduce constitutive increase of cAMP levels in F229V, I130N, and L312S variants but not in R137L/C [2,3,10,12,13]. In line with in vitro data, a patient carrying the R137L did not respond to the administration of these V2R antagonists [12].

Physiologically, vasopressin binds to the V2R expressed in the basolateral membrane of collecting duct principal cells and activates a Gs protein causing an increase in intracellular cAMP leading to a protein kinase A-mediated translocation of the Aquaporin-2 (AQP2) water channel to apical membrane of collecting duct principal cells thus promoting water reabsorption and urine concentration [14,15,16,17,18]. Recently, it has however been shown that vasopressin action may involve multiple distinct pathways to increase water reabsorption [19,20,21,22,23]. We have recently provided evidence that the functional consequences of expression of activating mutations R137L, R137C and F229V is a constitutive redistribution of the AQP2 water channel to the plasma membrane resulting in a significantly higher basal water permeability compared to V2R-wt expressing cells [13]. However, the basal levels of AQP2 phosphorylation at S256, which is critical for apical AQP2 accumulation but not for membrane association, were significantly higher in cells expressing the F229V mutant, but not in those expressing the R137L or R137C suggesting that while F229V mutant increases basal osmotic water through a cAMP-dependent pathway, R137L and R137C mutants might activate additional signaling pathways.

To better understand the intracellular pathways activated by the constitutively active R137L, R137C, and F229V mutants and how they might translate into the pathological outcome of NSIAD, in this study, we have investigated the phosphorylation pattern of AQP2 in cells expressing the wild-type V2R and its constitutively active mutants R137L, R137C and F229V. Our data provide novel evidence that R137L signals through an alternative PKA-independent pathway that increases AQP2 cell surface expression involving ROCK-induced phosphorylation of AQP2 at T269 independently of S256.

## 2. Materials and Methods

### 2.1. Chemicals and Reagents

Chemicals for Western blotting were from Bio-Rad (Bio-Rad Laboratories, Inc., Hercules, CA, USA). Calcein-AM was bought from Molecular Probes (Life Technologies, Monza, Italy). Cell culture media and FBS (fetal bovine serum) were from GIBCO (Life Technologies, Monza, Italy). Antibiotics were from Calbiochem. Super Signal^®^ West Pico Chemiluminescent Substrate (ThermoScientific, Rockford, IL, USA) and used for Chemidoc System (Bio-Rad Laboratories, Milan, Italy). All other chemicals were purchased from Merck (Merck KGaA, Darmstadt, Germany).

### 2.2. Antibodies

Aquaporin (AQP2) was detected using a specific antibody (C-tail Ab) raised against a synthetic peptide corresponding to the last 15 C-terminal amino-acids of human AQP2 [24]. Anti-phospho-AQP2 (Ser256) antibody was a kind gift from Peter Deen. Anti-phospho-AQP2 (Ser/Thr269) antibody was purchased from PhosphoSolutions (Aurora, Colorado). Anti-phospho-AQP2 (Ser261) antibody was from Novus Biologicals (Littleton, CO, USA). Anti-phospho-MYPT1 (Thr696) antibody was a component of the Rho-associated kinase (ROCK) Activity Assay purchased from Millipore (Merck KGaA, Darmstadt, Germany). Anti-Gα-13 was from Santa Cruz Biotechnology (Tebu Bio, Milan, Italy). Secondary goat anti-rabbit horseradish peroxidase–coupled antibodies were obtained from Merck (Merck KGaA, Darmstadt, Germany); secondary goat anti-mouse horseradish peroxidase–coupled antibodies were obtained from Bio-Rad (Bio-Rad Laboratories, Inc., Hercules, CA, USA).

### 2.3. Constructs

Human V2R wild type and mutants (R137L, R137C, F229V) with c-Myc epitope in N-terminal, expressed in pRK5 vector were a gift from Prof. Michel Bouvier (Université de Montréal, Montréal, Quebec, Canada). Constructs were obtained as previously reported [13]. Briefly, Human c-Myc-tagged V2Rs (wild type and mutants) were fused to the N-terminal of Rluc (Renilla luciferase) by linking each receptor sequence without its stop codon to Rluc cDNA through a 10-mer linker peptide (GGGGSGGGGS) and cloned into puromycin resistance retroviral expression vector pQCXIP (Clontech).

### 2.4. Cell Culture

Mouse cortical collecting duct MCD4 cells were stably transfected with the plasmid encoding the human AQP2 [25] and were engineered to permanently express chimeric V2R-Rluc using the pantropic retroviral expression system by Clontech as described in Ranieri et al. [13]. Briefly, recombinant retroviruses expressing receptor–Rluc fusion proteins were prepared by transfection of GP2-293 packaging cell with different retroviral vectors using polyethyleneimine linear MW 25,000 Da (PEI). Cells were allowed to increase the viral titer for 48 h before collecting the virus-containing supernatants. MCD4 cells were infected with the V2R-Rluc retroviruses in the presence of 8 μg/mL polybrene for 24 h and selected under puromycin (1 µg/mL).

Then MCD4 were grown in a 1:1 mixture of Dulbecco’s modified Eagle’s medium and F-12 supplemented with 5% (*v*/*v*) fetal bovine serum (Thermo Fisher Scientific, Waltham, MA, USA), 1% (*v*/*v*) L-glutamine (Thermo Fisher Scientific, Waltham, MA, USA) and 100 IU/mL penicillin, 100 µg/mL streptomycin (Euroclone, Milan, Italy), 5 µM dexamethasone (Merck KGaA, Darmstadt, Germany), 400 µg/mL G418 (for AQP2 resistance) (Thermo Fisher Scientific, Waltham, MA, USA) and 1 µg/mL puromycin (for V2R-Rluc resistance) (Thermo Fisher Scientific, Waltham, MA, USA), in a humidified atmosphere of 5% CO_2_ at 37 °C.

### 2.5. Gel Electrophoresis and Immunoblotting

For immunoblotting studies, cells grown on 60 mm dishes were lysed in Cell Fractionation Buffer (20 mM NaCl, 130 mM KCl, 1 mM MgCl2, 10 mM Hepes, pH 7.5) in the presence of proteases (1 mM PMSF, 2 mg/mL leupeptin, and 2 mg/mL pepstatin A) and phosphatases (10 mM NaF and 1 mM sodium orthovanadate) inhibitors. After sonication (60 kHz for 5 s), lysates were centrifuged at 12,000× *g* for 10 min. The supernatants were collected and used for immunoblotting studies.

Proteins were separated on 12% stain-free polyacrylamide gels (Bio-Rad Laboratories, Inc., Hercules, CA, USA) under reducing conditions. Protein bands were electrophoretically transferred onto Immobilon-P membranes (Merck KGaA, Darmstadt, Germany) for Western blot analysis, blocked in TBS-Tween-20 containing 3% bovine serum albumin (BSA) and incubated with primary antibodies O/N (anti-AQP2, anti-AQP2-pS256, -pS269 and-pS261, anti-MYPT1-T696, and anti-Gα-13). Immunoreactive bands were detected with secondary goat anti-mouse horseradish peroxidase–coupled antibodies. Membranes were incubated with Super SignalWest Pico Chemiluminescent Substrates (Thermo Fisher Scientific, Waltham, MA, USA), and the signals were visualized with the ChemiDoc System gels (Bio-Rad Laboratories, Inc., Hercules, CA, USA). Obtained bands were normalized to total protein using the stain-free technology gels (Bio-Rad Laboratories, Inc., Hercules, CA, USA). Densitometry analysis was performed using Image Lab gels (Bio-Rad Laboratories, Inc., Hercules, CA, USA). Data were analyzed using GraphPad Prism (GraphPad Prism Software 8.0.1, San Diego, CA, USA).

### 2.6. Water Permeability Assay

Osmotic water permeability was measured by Video Imaging experiments as previously described [26]. Briefly, MCD4 cells were grown onto 40 mm glass coverslips and loaded with 10 μM membrane-permeable Calcein-AM for 45 min at 37 °C, 5% CO_2_ in DMEM. Cells were left under basal condition or stimulated with 100 nM desmopressin (dDAVP) for 45 min. When indicated, cells were pretreated with the specific Protein Kinase Inhibitor (PKI) at 10 µM for 30 min or with the specific Rho Kinase Inhibitor (Y27632) at 100 µM for 30 min under basal conditions or before dDAVP stimulation. The coverslips with dye-loaded cells were mounted in a perfusion chamber (FCS2 Closed Chamber System, BIOPTECHS, Butler, PA, USA) and measurements were performed using an inverted microscope (Nikon Eclipse TE2000-S microscope) equipped for single-cell fluorescence measurements and imaging analysis. The Calcein-AM loaded sample was excited at 490 nm. Fluorescence measurements, following iso-(140 mM NaCl, 5 mM KCl, 1 mM MgCl_2_, 1 mM CaCl_2_, 10 mM Hepes sulfonic acid, 5 mM Glucose, pH 7.4) or hyperosmotic (isosmotic solution added with 135 mM Mannitol) solutions, were carried out using Metafluor Software 7.8.1.0 (Molecular Devices, LLC, San Jose, CA, USA). The time course of cell shrinkage was measured as a time constant (K_i_ or 1/tau expressed in sec^−1^), a parameter directly correlated to membrane water permeability.

### 2.7. Fluorescence Resonance Energy Transfer Measurements

To evaluate the basal activity of RhoA, fluorescence resonance energy transfer (FRET) experiments were performed. Briefly, MCD4 cells were seeded onto 20-mm glass coverslips at 37 °C, 5% CO2, and transiently transfected with a plasmid encoding the ECFP-Raichu-RBD-EYFP. Experiments were performed 48 h after transfection and cells were left under basal condition or stimulated with the Rho proteins inhibitor C3 toxin at 1 µg/mL for 3 h, used as an internal control. Raichu-RBD contains YFP and CFP separated by rhotekin-RBD (RBD). Active Rho-GTP binds RBD, separating the donor (CFP) from the acceptor (YFP) thus reducing FRET. Visualization of ECFP- and/or EYFP-expressing cells and detection of FRET was performed on an inverted microscope (Nikon Eclipse TE2000-S), equipped with a monochromator controlled by Metamorph^®^ Microscopy Automation and Image Analysis Software 7.8.1.0 (Molecular Devices, LLC, San Jose, CA, USA). ECFP was excited at 436 nm and EYFP at 500 nm. All images were aligned and corrected for background in the emission windows for FRET (535/30 nm), ECFP (475/30 nm), and EYFP (535/26 nm). Each image was further corrected for ECFP crosstalk and EYFP cross-excitation as shown by Russo A [27].

Thus, netFRET = IFRETbg − ICFPbg × K1-IYFPbg (K2-αK1)]/(1-δK1), where IFRETbg, ICFPbg, and IYFPbg are the background-corrected pixel grey values measured in the FRET, ECFP, and EYFP windows, respectively; K1, K2, α, and δ are calculated to evaluate the crosstalk between donor and acceptor. The obtained netFRET values were normalized for the expression levels of ECFP and EYFP (NFRET = netFRET × 100/(ICFPbg × IYFPbg)1/2). The integrated fluorescence density values of the images from ten regions of interest in each cell were analyzed using MetaMorph Software 7.8.1.0 (Molecular Devices, LLC, San Jose, CA, USA) and Microsoft Excel Software (Office 365, Microsoft Corporation, Redmond, WA, USA).

### 2.8. F-actin co-Sedimentation Assay

F-actin co-sedimentation was performed as described previously [27]. Briefly, total membrane and cytosol fractions were prepared from MCD4-V2R-wt and mutant cells. Cells were scraped and resuspended in homogenization buffer that contained 20 mM Tris-HCl (pH 8.0), 1 mM EDTA, 1 mM dithiothreitol and protease inhibitors (1 mM PMSF, 2 mg/mL leupeptin, and 2 mg/mL pepstatin A). Cells were homogenized using a 27-gauge needle, and nuclei were removed by centrifugation at 800× *g* for 10 min. Membrane and cytosol fractions were obtained by centrifugation for 1 h at 4 °C at 150,000× *g*. Cytosolic proteins (800 μg each condition) were used for F-actin polymerization. Specifically, the formation of F-actin was initiated using a 50-fold polymerization buffer that contained 200 mM MgCl2, 4 M KCl, and 100 mM ATP. The samples were incubated for 1 h at 37 °C, and F-actin was pelleted by ultracentrifugation for 1 h at 4 °C at 150,000× *g*. The F-actin-containing pellets were rinsed with a homogenization buffer. Cytosol and F-actin fractions were separated by 13% SDS-PAGE and immunoblotted with G-alpha-13 specific antibodies.

### 2.9. Rho-Associated kinase (ROCK) Activity Assay

ROCK activity was measured with a Rho-associated kinase activity assay Kit from Millipore (Merck KGaA, Darmstadt, Germany) according to the manufacturer’s protocol with certain modifications. Both ROCK1 and ROCK2 activity were tested obtaining comparable results therefore we generally refer to ROCK activity in the results. MCD4 cells were lysed with RIPA buffer in the presence of protease (1 mM PMSF, 2 mg/mL leupeptin, and 2 mg/mL pepstatin A) and phosphatases (10 mM NaF and 1 mM sodium orthovanadate) inhibitors. Lysates were centrifuged at 12,000× *g* for 10 min and 50 μL of the supernatants were deposited in 96-well multi-strip plates pre-coated with MYPT1 supplied with 10 mM DTT, 2 mM MgCl2 and 10 mM ATP for 60 min at 30 °C. Anti-phospho-MYPT1 (Thr696) antibody was then added for 1 h at room temperature. Goat anti-rabbit IgG HRP secondary antibody was added for another 1 h at room temperature, and chromogenic substrate tetramethylbenzidine (TMB) was added for 15 min. Absorbance at 450 nm reflected the relative amount of ROCK activity in the sample, which was evaluated relative to the total protein content of each sample and was read using a microplate reader from Bio-Rad (Bio-Rad Laboratories, Inc., Hercules, CA, USA). Data were analyzed using GraphPad Prism (GraphPad Prism Software 8.0.1, San Diego, CA, USA).

### 2.10. In Vitro Phosphorylation of Synthetic Peptides

In total, 8 µg of a synthetic unmodified AQP2 carboxyl-terminal peptide (VELHSPQSLPRGTKA) (Primm Srl, Milan, Italy) were incubated at 30 °C for 1 h in phosphorylation buffer (0.1 mM ATP, 50 mM Tris pH = 7.5, 10 mM MgCl_2_) in the presence of 400 ng of ROCK1 (Thermo Fisher Scientific, MA, USA) or ROCK2 (Merck KGaA, Darmstadt, Germany) kinases. In parallel, 8 µg of unmodified AQP2 were incubated with 400 ng of ROCK1 or ROCK2 in the presence of 100 μM of ROCK inhibitor Y27632. As a control, unmodified AQP2 peptide and the same peptide pre-phosphorylated at threonine-269 (VELHSPQSLPRG(pT)KA) (Primm Srl, Milan, Italy) were used, respectively [28]. Preliminary dose-response experiments were performed to choose the concentration of ROCK and Y27632 used.

To evaluate whether ROCK can phosphorylate in vitro the AQP2 peptide, the dot-blot technique was used. Specifically, 12 μL of each reaction were deposited onto Immobilon-P membranes (Merck KGaA, Darmstadt, Germany). When the membrane was completely dry, it was activated and blocked in TBS–Tween-20 containing 3% bovine serum albumin (BSA) and incubated with anti-AQP-pS269 (PhosphoSolutions, Aurora, CO) overnight. Immunoreactive spots were detected with secondary goat anti-rabbit horseradish peroxidase-coupled antibodies. Membranes were incubated with Super Signal West Pico Chemiluminescent Substrates (Thermo Fisher Scientific, MA, USA) and the signals were visualized with the ChemiDoc System gels (Bio-Rad Laboratories, Inc. CA, USA). Comparable results were obtained with both ROCK1 and ROCK2 therefore we generally refer to ROCK in the results.

### 2.11. Statistical Analysis

One-way ANOVA followed by Newman–Keuls multiple comparisons with each column was used for the statistical analysis. All values are expressed as means ± SEMs. A difference of *p*<0.05 was considered statistically significant.

## 3. Results

### 3.1. V2R R137L and R137C expressing cells have significantly higher basal levels of pT269-AQP2

**Figure 1:**
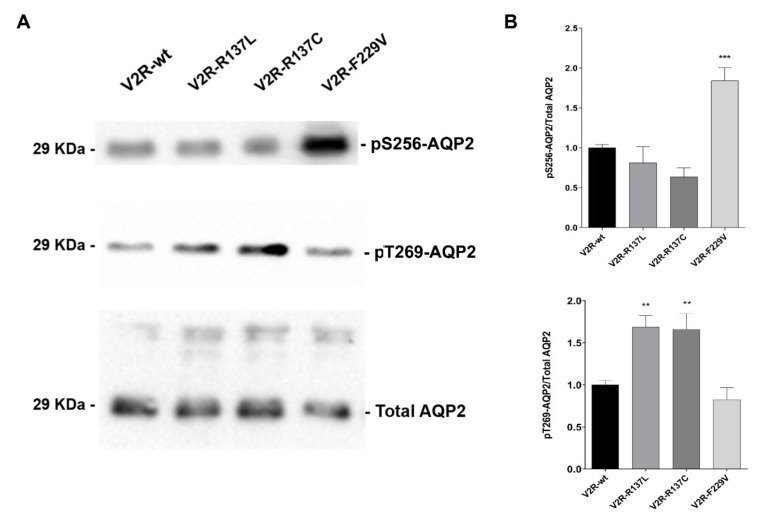
V2R Cells expressing R137L and R137C have significantly higher basal levels of p269-AQP2. (**A**). Representative western blots of MCD4 lysates showing phosphorylation of AQP2 at Ser-256 (pS256-AQP2) or Thr-269 (pT269-AQP2) in cells expressing the wild-type V2R or the constitutively active R137L, R137C and F229V under basal conditions. For normalization, cell lysates were also probed for total AQP2 (Total AQP2). (**B**). Densitometric analysis of pS256-AQP2 and pT269-AQP2 bands normalized to total AQP2 bands (Total AQP2) is reported in the histograms. The data are means (±S.E.M.) of 5 experiments (** *p* < 0.01 or *** *p* < 0.001 vs. V2R-wt).

We have previously provided evidence of a direct link between the expression of constitutively active V2R mutants and increased cell surface expression of AQP2 and osmotic water permeability in MCD4 cells under basal conditions [13]. Moreover, while the V2R-F229V mutant seemed to cause constitutive AQP2 relocation in a cAMP/PKA dependent pathway, the V2R-R137L and R137C mutants seemed to increase AQP2 targeting to the plasma membrane independently of cAMP increase [13]. Therefore, the profile of basal levels of AQP2 phosphorylation at different sites was evaluated in cells expressing the V2R-F229V, R137L, and R137C mutants by western blotting using phosphospecific antibodies (Figure 1A,B).

Compared to wild-type V2R, in cells expressing V2R-R137L and R137C, no changes in basal levels of pS256-AQP2 where observed (V2R-R137L: 0.81 ± 0.20; V2R-R137C: 0.64 ± 0.11), whereas those expressing V2R-F229V had significantly higher levels of pS256-AQP2 (V2R-F229V: 1.84 ± 0.17; *p* < 0.001 vs V2R-wt: 1.00 ± 0.04) thus confirming our previous finding [13]. Interestingly, the analysis of pT269-AQP2 levels in cells expressing the active R137L and R137C mutants, showed a mirror situation, with significantly higher levels of pT269-AQP2 (V2R-R137L: 1.69 ± 0.14; V2R-R137C: 1.66 ± 0.19; *p* < 0.01 vs. V2R-wt: 1.00 ± 0.06) while in F229V, pT269-AQP2 was unaffected (V2R-F229V: 0.82 ± 0.14) (Figure 1A,B). The pS261-AQP2 basal levels in cells expressing the V2R mutants were similar compared to V2R-wt expressing cells (data not shown).

These data suggest that in cells expressing the V2R-F229V mutant, constitutive AQP2 translocation to the apical membrane involves cAMP/PKA-dependent phosphorylation of S256. In contrast, constitutive AQP2 relocation to the plasma membrane in V2R-R137L or R137C mutants involves a pathway leading to AQP2 phosphorylation at T269, known to be important to retain AQP2 in the plasma membrane [28,29].

### 3.2. Sensitivity of Osmotic Water Permeability to PKA Inhibitor.

**Figure 2:**
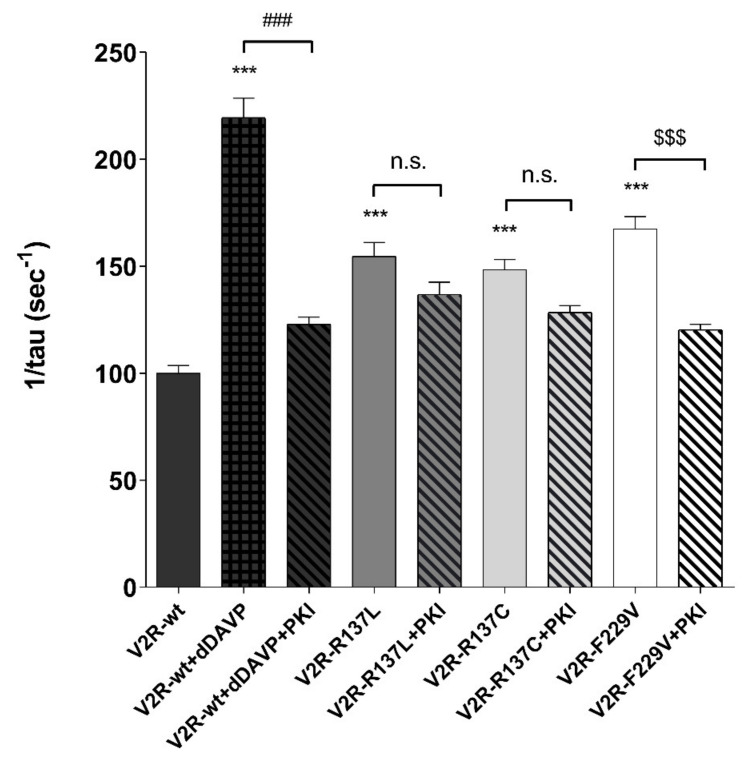
Effect of the PKA inhibitor, PKI, on osmotic water permeability in MCD4 cells expressing wild type and mutated V2R. The temporal osmotic response is indicated as 1/tau (expressed in sec^−1^). In cells expressing the wild-type V2R, PKA inhibitor, PKI (10 µM for 30 min), prevented the increase in the osmotic water permeability in response to desmopressin (dDAVP, 100 nM for 45 min). In cells expressing the active mutants, the PKA inhibitor, PKI (10 µM for 30 min), significantly reduced the basal osmotic water permeability only in F229V expressing cells. The data, expressed as a percentage of the values measured in the cell expressing the wild-type V2R, are means (±S.E.M.) of 5 experiments (*** *p* < 0.001 vs. V2R-wt; ### *p* < 0.001 vs. V2R-wt+dDAVP; $$$ *p* < 0.001 vs. V2R-F229V; n.s.: not significant vs V2R-R137L or V2R-R137C).

To explore the relevance of the cAMP/PKA pathway in AQP2 translocation for all V2R variants, the osmotic water permeability was measured in MCD4 cells expressing either the V2R-wt or different receptor mutants in the presence or the absence of a specific inhibitor of the PKA, PKI. The basal temporal osmotic response in V2R mutants (V2R-R137L: 154.70 ± 6.48%; V2R-R137C: 148.40 ± 4.81%; V2R-F229V: 167.50 ± 5.80%; *p* < 0.001 vs. V2R-wt: 100.00 ± 3.60%) was significantly higher compared with that of cells expressing the wild-type V2R. Preincubation with PKI reduced the basal osmotic water permeability only in cells expressing the F229V mutant (V2R-F229V+PKI: 120.3 ± 2.64%; *p* < 0.001 vs. V2R-F229V), whereas had no effect in those expressing the R137L (V2R-R137L+PKI: 136.80 ± 5.85%) and R137C (V2R-R137C+PKI: 128.40 ± 3.18%) mutants (Figure 2).

As expected, PKI prevented the dDAVP-induced increase in the osmotic water permeability in V2R-wt expressing cells (V2R-wt+dDAVP+PKI: 122.90 ± 3.35%; *p* < 0.001 vs. V2R-wt+dDAVP: 219.50 ± 9.16%) (Figure 2). These findings confirm, as previously shown, a direct link between constitutively activating mutations of the V2R and increased water reabsorption in kidney cells under basal conditions. However, they provide evidence of an alternative PKA-independent pathway that increases AQP2 membrane targeting and osmotic water permeability in cells expressing the R137C and R137L mutants which involves an increase in pT269-AQP2, independently of pS256 phosphorylation.

### 3.3. R137L and R137C Mutants Activate a Signaling Pathway Involving Rho-Associated Kinase (ROCK)

S/T269 phosphorylation is thought to be important for AQP2 membrane retention [28,29], however, the kinase involved remains elusive. In vitro studies have shown that PKA does not phosphorylate S/T269 [28]. Since S/T269 lies within a ROCK phosphorylation motif RXS/T [22], we first tested whether ROCK could phosphorylate, *in vitro,* a peptide reproducing the AQP2 C-terminus.

**Figure 3:**
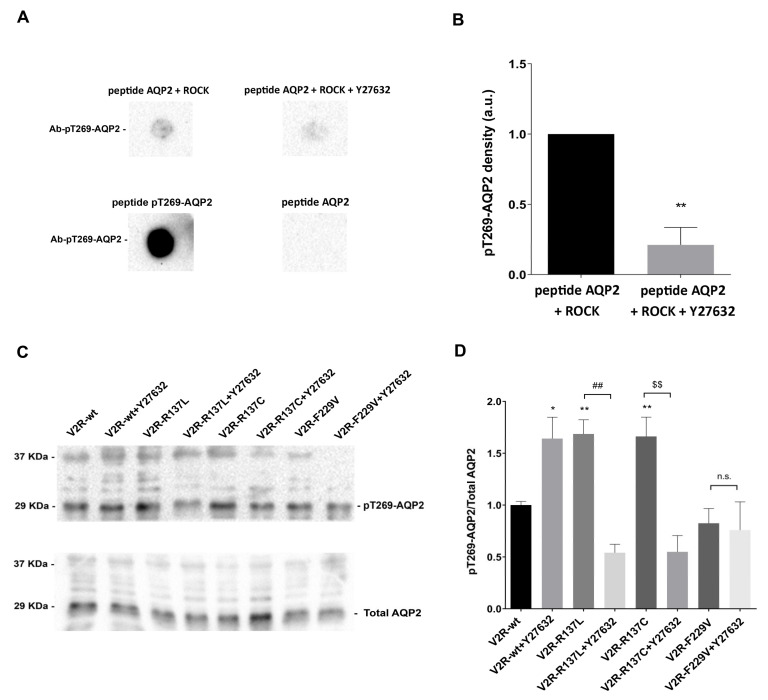
In vitro phosphorylation of AQP2 peptide at Thr-269 by ROCK and effect of ROCK inhibition on pT269-AQP2 levels in V2R mutants. (**A**). Phosphorylation of the AQP2 peptide was assessed by the dot-blot technique. A representative dot-blot experiment using the synthetic peptide (VELHSPQSLPRG(pT)KA) is shown. As a negative and positive control, unmodified AQP2 peptide or the same peptide pre-phosphorylated at threonine-269 were used, respectively. Unmodified AQP2 peptide was incubated with ROCK (400 ng) in the presence or absence of the ROCK inhibitor Y27632 (100 μM). (**B**). Densitometric analysis of pT269-AQP2 dots (a.u., arbitrary units) is reported in the histogram. The data are means (±S.E.M.) of 3 experiments (** *p* < 0.01 vs peptide AQP2 + ROCK). (**C**). Representative western blots of MCD4 lysates showing phosphorylation of AQP2 at Thr-269 (pT269-AQP2) in cells expressing the wild-type V2R or the constitutively active R137L, R137C and F229V under basal conditions in the presence or the absence of the ROCK inhibitor Y27632 (100 μM for 30 min). For normalization, cell lysates were also probed for total AQP2 (Total AQP2). (**D**). Densitometric analysis of pT269-AQP2 bands normalized to total AQP2 bands (Total AQP2) is reported in the histogram. The data are means (±S.E.M.) of 5 experiments (* *p* < 0.05 or ** *p* < 0.01 vs. V2R-wt; ## *p* < 0.01 vs. V2R-R137L; $$ *p* < 0.01 vs. V2R-R137C; n.s.: not significant vs. V2R-F229V).

To this end, a synthetic peptide including 15 amino acids of AQP2 carboxyl-terminal (VELHSPQSLPRGTKA) was subjected to in vitro phosphorylation using purified ROCK, in the presence and the absence of the specific ROCK inhibitor Y27632. The reaction was performed as described in Methods. The results of these experiments show that ROCK can phosphorylate in vitro the AQP2 peptide at T269 and that phosphorylation was almost (80%) completely prevented in the presence of the ROCK inhibitor Y27632 (Figure 3A,B).

The ability of the anti-AQP2-pT269 in recognizing the T269 site in the AQP2 peptide was confirmed by testing the immunoreactivity of the anti-AQP-pT269 antibody on the AQP2 peptide pre-phosphorylated at T269 (VELHSPQSLPRG(pT)KA) (Figure 3A). Conversely, the anti-AQP2-pT269 antibody did not detect the unmodified AQP2 peptide (Figure 3A).

To evaluate whether ROCK is responsible for the high pT269-AQP2 levels in living cells expressing V2R-R137L and V2R-R137C, the effect of ROCK inhibitor Y27632 was tested. pT269-AQP2 levels in cells expressing V2R variants were measured in the presence and the absence of ROCK inhibitor Y27632 using a phosphospecific antibody. Interestingly, pre-treatment with Y27632 strongly reduced pT269-AQP2 levels both in V2R-R137L (V2R-R137L + Y27632: 0.54 ± 0.08; *p* < 0.01 vs. V2R-R137L: 1.69 ± 0.14) and V2R-R137C (V2R-R137C + Y27632: 0.55 ± 0.16; *p* < 0.01 vs. V2R-R137C: 1.66 ± 0.19) expressing cells (Figure 3C,D). In contrast, the ROCK inhibitor had no effect on F229V expressing cells. Of note, in V2R-wt, pretreatment with Y27632 induced instead a significant increase in pT269-AQP2 levels (V2R-wt + Y27632: 1.64 ± 0.21; *p* < 0.05 vs. V2R-wt: 1.00 ± 0.04). These results lead to two main conclusions: a. in cells expressing V2R-R137L and R137C mutants AQP2 phosphorylation at T269 is mediated by ROCK; b. since in cells expressing the V2R-wt inhibition of ROCK induces an increase in pT269-AQP2 levels, two different signalosomes can regulate pT269-AQP2, i.e., in V2R-R137L and R137C expressing cells a pathway causing ROCK-dependent pT269-AQP2, whereas, in cells expressing the V2R-wt, a signalosome involving ROCK inhibition. Y27632 did not affect F229V phosphorylation further confirming that active mutants have differential effects on the AQP2 phosphorylation profile (Figure 3C,D).

**Figure 4:**
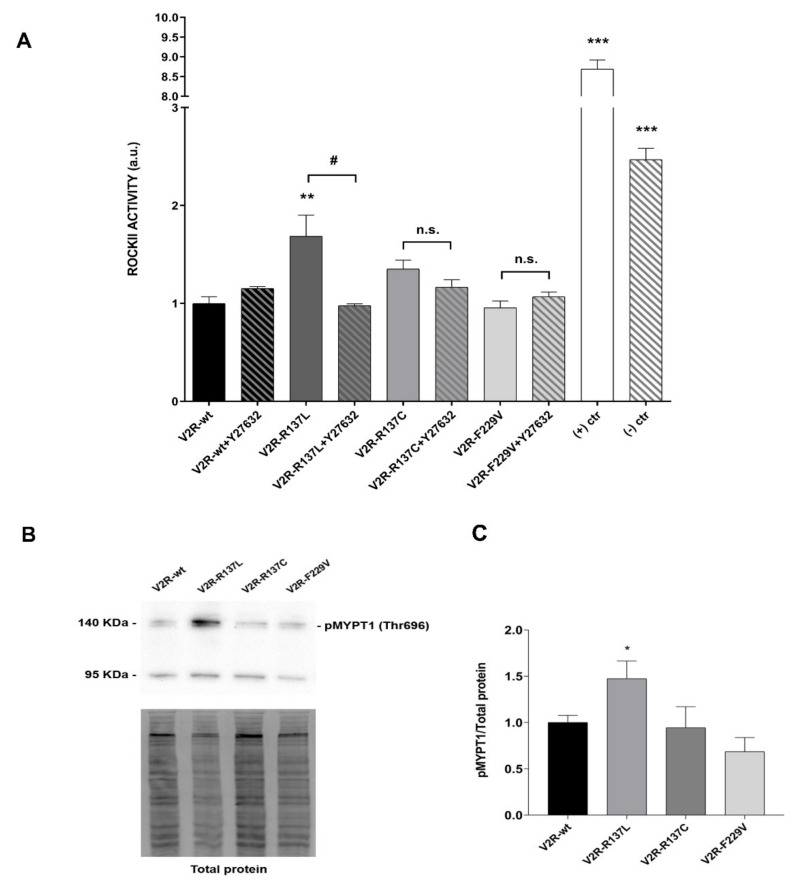
ROCK activity in MCD4 cells expressing wild type and mutated V2R. (**A**). ROCK activity in cells expressing the wild-type V2R or the constitutively active R137L, R137C and F229V under basal conditions in the presence or the absence of the ROCK inhibitor Y27632 (1 mM), was tested using a ROCK activity assay Kit as described in “Materials and methods”. V2R-R137L mutant displayed a significantly higher ROCK activity sensitive to Y27632. The data are normalized compared to the activity measured in V2R-wt MCD4 expressing cells and are represented as the means (± S.E.M.) of 3 independent experiments. (** *p* < 0.01 or *** *p* < 0.001 vs. V2R-wt; # *p* < 0.05 vs. V2R-R137L; n.s.: not significant vs. V2R-R137C or V2R-F229V). The histogram includes the activity of ROCK enzyme at 1 mU/µL, in the absence (positive control (+) ctr), or in the presence (negative control (-) ctr), of the ROCK inhibitor, Y27632 (1 mM). (**B**)**.** Representative western blot showing ROCK activity by monitoring phosphorylation of MYPT1 on Thr-696 (pT696-MYPT1), a downstream protein substrate of ROCK, in cells expressing the wild-type V2R or the constitutively active R137L, R137C and F229V under basal conditions. The intensity of MYPT1 bands was normalized to total protein (see “Materials and methods” for stain-free technique). (**C**)**.** Densitometric analysis of pT696-MYPT1 bands normalized to total protein. The data, normalized to wild-typeV2R, are expressed as means (± S.E.M.) of 5 experiments (* *p* < 0.05 vs. V2R-wt).

We next evaluated the activity of the enzyme ROCK using a specific ELISA test. The results of these experiments revealed that, compared to V2R-wt, the only mutant displaying a significantly higher basal ROCK activity, is V2R-R137L (V2R-R137L: 1.69 ± 0.22; *p <* 0.01 vs. V2R-wt: 1.00 ± 0.07) (Figure 4A).

In the V2R-R137L expressing cells, ROCK activity was sensitive to the ROCK inhibitor Y27632 which reduced the basal ROCK activity measured in V2R-R137L (V2R-R137L+Y27632: 0.98 ± 0.02; *p <* 0.05 vs. V2R-R137L: 1.69 ± 0.22). In V2R-R137C a tendency of a higher ROCK activity was observed in basal conditions, but it did not reach a statistical significance compared to that measured in the V2R-wt expressing cells (Figure 4A).

The activity of the enzyme ROCK was further evaluated by monitoring the phosphorylation of the downstream protein substrate MYosin Phosphatase Target 1 (MYPT1) in Thr696. In line with the ELISA data, V2R-R137L expressing cells displayed a significant increase in pT696-MYPT1 phosphorylation compared to V2R-wt cells (V2R-R137L: 1.47 ± 0.19; *p <* 0.05 vs. V2R-wt: 1.00 ± 0.08) (Figure 4B,C).

### 3.4. Gα12/13–Rho–ROCK Signaling is Activated by the V2R-R137L Mutant

To determine the underlying mechanism leading to ROCK activation, we hypothesize Gα12/13–Rho–ROCK signaling as the pivotal mechanism involved.

**Figure 5:**
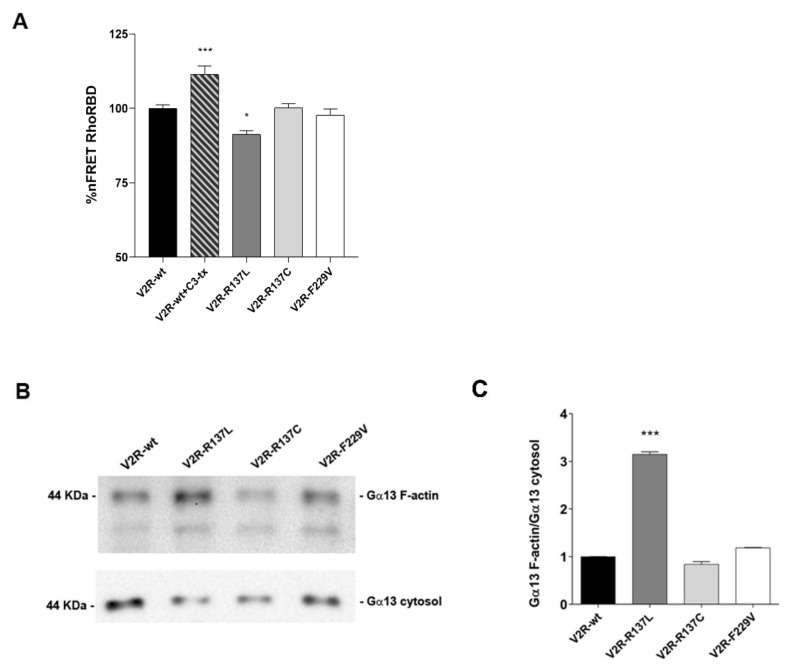
Evaluation of Rho and Gα13 activity in MCD4 cells expressing wild type and mutated V2R. (**A**)**.** Evaluation of Rho activity by Fluorescence Resonance Energy Transfer (FRET) experiments. MCD4 cells expressing wild-type V2R and V2R mutants were transiently transfected with Raichiu-RBD as described in the “Materials and methods” section. Histogram reports nFRET measured in cells expressing the constitutively active R137L, R137C, and F229V mutants under basal conditions. The R137L mutants displayed a significantly higher Rho activity depicted as a reduced nFRET signal. As internal control the Rho activity was tested in wild-type V2R left under basal condition or pretreated with the C3 toxin (1µg/mL for 3 h), an inhibitor of Rho GTPase proteins (* *p* < 0.05 or *** *p* < 0.001 vs. V2R-wt). (**B**)**.** Evaluation of Gα13 activity by F-actin co-sedimentation assay. Representative western blot showing Gα13 expression under basal conditions in F-actin polymerized fraction and in the cytosolic soluble fraction in MCD4 cells expressing wild-type or V2R mutants. (**C**). Densitometric analysis of Gα13 bands in F-actin enriched fraction normalized to a cytosolic soluble fraction is reported in the histogram. Relative to the V2R-wt, a significant increase in immunodetectable Gα13 was observed in the F-actin–enriched only in V2R-R137L indicating a significantly higher Gα13 activity in V2R-R137L. The data are means (±S.E.M.) of 5 experiments (*** *p* < 0.001 vs. V2R-wt).

Since ROCK is a downstream effector of RhoA, the basal activity of RhoA was measured in cells expressing V2R mutants by Fluorescence Resonance Energy Transfer (FRET). The Raichu-RBD probe, consisting of Venus and cyan fluorescent protein (CFP) moieties separated by rhotekin-RBD, was used, as previously described [30]. Activation of RhoA results in binding to rhotekin-RBD, increased separation of the fluorophores, and consequent loss of FRET. In contrast, Rho inhibition promotes the transfer of fluorescence from the donor (CFP) to the acceptor (YFP) thus increasing the signal of FRET. Compared to V2R-wt expressing cells, the basal activity of RhoA was significantly higher only in V2R-R137L (V2R-R137L: 91.23 ± 1.29%; *p <* 0.05 vs. V2R-wt: 100.00 ± 1.19%) (Figure 5A).

The specificity of the probe was confirmed by the increased FRET signal obtained in V2R-wt cells treated with C3 toxin which inactivates Rho proteins (V2R-wt+Cr-tx: 111.40 ± 2.93%; *p <* 0.001 vs. V2R-wt) (Figure 5A).

The heterotrimeric G proteins Gα12 and Gα13 have been described as RhoA activators via RH-containing RhoGEFs [31,32].

To evaluate Gα13 activity, F-actin co-sedimentation was performed as described previously [33]. The affinity between F-actin and Gα13 might be considered an important indicator of Gα13 activity [34]. Cytosolic fractions of V2R variant expressing cells were prepared, F-actin polymerization was induced, and F-actin interacting proteins were analyzed by Western blotting. Relative to the V2R-wt, a significant increase in immunodetectable Gα13 in the F-actin–enriched fraction was observed only in V2R-R137L (V2R-R137L: 3.15 ± 0.05; *p* < 0.001 vs. V2R-wt: 1.00 ± 0.001) (Figure 5B,C). In contrast, the amount of Gα13 that co-sedimented with the F-actin fraction in V2R-R137C (0.84 ± 0.04) and V2R-F229V (1.19 ± 0.01) was comparable to that observed in V2R-wt cells (Figure 5B,C). Together these data suggest that Gα12/13–Rho–ROCK signaling is involved in the V2R-R137L pathway.

### 3.5. Inhibition of ROCK Reduces the Basal Osmotic Water Permeability in V2R-R137L Expressing Cells

**Figure 6:**
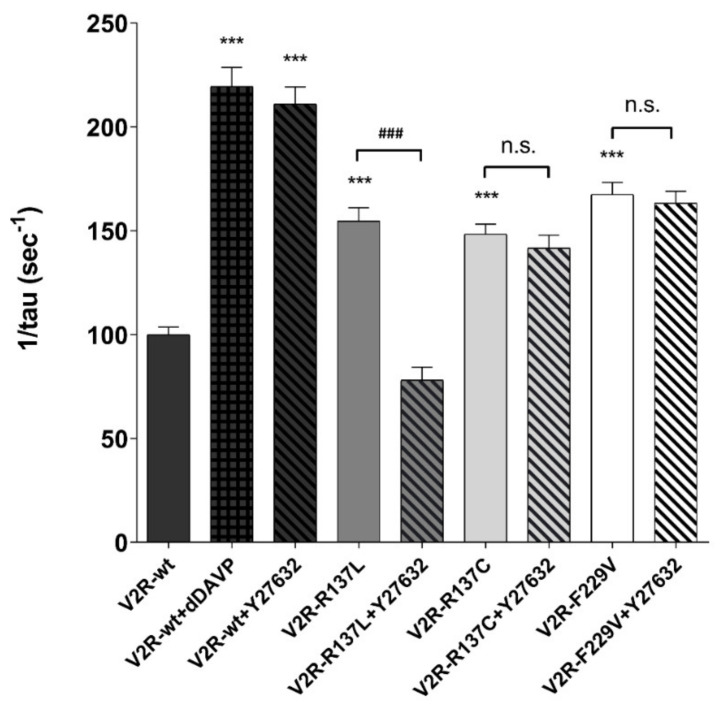
ROCK inhibition reduces the basal osmotic water permeability in V2R-R137L expressing cells. Osmotic water permeability studies in MCD4 cells expressing the wild-type V2R and constitutive active R137L, R137C, and F229V were performed as reported in “Materials and methods”. The temporal osmotic response is indicated as 1/tau (expressed as sec^−1^). In cells expressing all constitutive active mutants, the temporal osmotic response was significantly higher compared with cells expressing the wild-type V2R. The V2R-R137L mutant was sensitive to the ROCK inhibitor Y27632 displaying a significant inhibition in the osmotic response. In V2R-wt expressing cells, dDAVP stimulation significantly increased the osmotic water permeability. Exposure of V2R-wt cells to Y27632 increased the osmotic water permeability in the absence of hormonal stimulation. The data are means (± S.E.M.) of 5 experiments. (*** *p* < 0.001 vs. V2R-wt; ### *p <* 0.001 vs. V2R-R137L; n.s.: not significant vs. V2R-R137C or V2R-F229V).

We have previously demonstrated that, in cells expressing the constitutively active mutants, the temporal osmotic response was significantly higher compared with that of cells expressing the wild-type V2R [13], indicating increased water permeability in the absence of dDAVP stimulation and the increase in water permeability correlates with AQP2 translocation. To assess the functional outcome of ROCK inhibition on the osmotic response, the osmotic water permeability was measured in MCD4 cells expressing either the wild-type V2R and the different receptor mutants. As shown in Figure 6, in cells expressing all constitutive active mutants, the temporal osmotic response was significantly higher compared with cells expressing the wild-type V2R (V2R-R137L: 154.70 ± 6.48%; V2R-R137C: 148.40 ± 4.81%; V2R-F229V: 167.50 ± 5.80%; *p <* 0.001 vs. V2R-wt: 100.00 ± 3.60%) and only the V2R-R137L mutant was sensitive to the ROCK inhibitor Y27632 displaying a significant inhibition in the osmotic response (V2R-R137L+Y27632: 78.10 ± 6.2%; *p <* 0.001 vs. V2R-R137L: 154.70 ± 6.48%). As expected, dDAVP stimulation significantly increased the osmotic water permeability (V2R-wt+dDVAP: 219.50 ± 9.16%; *p <* 0.001 vs. V2R-wt) confirming that these cells express a functional V2R-wt. The sole exposure of V2R-wt cells to Y27632 increased the osmotic water permeability in the absence of hormonal stimulation in agreement with previous findings [35].

## 4. Discussion

In the present study, we identified a novel signaling pathway associated with rare gain-of-function variants of V2R causing NSIAD. The major results can be summarized as follows: *a*. active V2R-R137L and R137C mutants have significantly higher basal pT269-AQP2 levels -independently of S256 and PKA-which were reduced to control by treatment with ROCK inhibitor; *b*. ROCK activity was found significantly higher in V2R-R137L, with a tendency for a higher activity also in V2R-R137C; *c.* the Gα12/13–Rho–ROCK pathway was found activated in the V2R-R137L expressing cells; *d.* the basal elevated osmotic water permeability in the V2R-R137L mutant was reduced upon inhibition of ROCK.

The V2R-R137L mutation affects a conserved DRY/H motif in the second intracellular loop on the vasopressin receptor [1,2], whereas the mutation V2R-F229V is located in the third intracellular loop of V2R [2]. Evidence has been provided that the DRY/H motif plays a crucial role in the stabilization of the receptor in its constitutive active or inactive form [5]. In fact, the substitution of the arginine for histidine (R137H) causes a loss of function of the V2R resulting in the mirror disease of NSIAD, NDI. By structural modeling, it has been shown that V2R-R137L mutation disrupts an interaction with S/T269 residues in the V2R that switch the receptor in an activated form independent of vasopressin binding [36].

We show here that, while all three mutations tested (R137L, R137C, and F229V) have constitutive elevated osmotic water permeability, specific inhibition of PKA does not affect the basal osmotic water permeability in V2R-R137L and R137C mutants, whereas it does in V2R-F229V mutant expressing cells (Figure 2). This suggests that the R137L/C mutation may activate alternative PKA-independent signaling to cause AQP2 insertion to the plasma membrane. Another important implication of our study is that S256 phosphorylation is not a pre-requisite for S/T269 phosphorylation in R137L/C. Previous reports showed that phosphorylation at S256 precedes phosphorylation of other serine residues within the AQP2 C-terminus [37,38]. However, subsequent studies suggested that the phosphorylation cascade of these serine residues is not sequential as originally thought [28]. Among these serines, S269 phosphorylation is thought to be crucial for AQP2 membrane retention [28,29] and recent studies highlight the importance of S269 phosphorylation in AQP2 trafficking independently of S256 phosphorylation.

**Figure 7:**
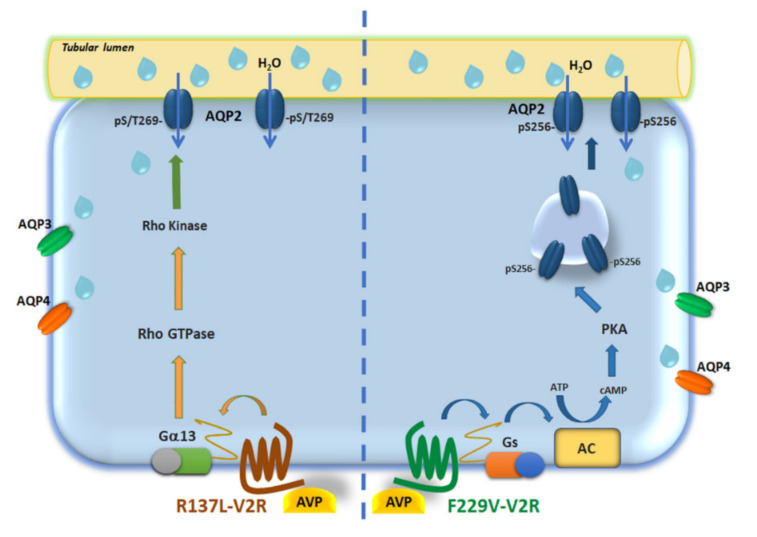
Schematic model depicting two distinct signalings activated by the gain of function V2R-F229V and V2R-R137L causing NSIAD: The gain-of-function mutation R137L signals through an alternative PKA-independent pathway that increases AQP2 membrane targeting through ROCK induced phosphorylation at S/T269 independently of S256 of AQP2. Conversely, V2R-F229V expression overactivated the cAMP/PKA axis with a consequent increase in PKA-dependent pS256-AQP2.

In cultured AQP2-expressing cells and kidney collecting duct principal cells, it has been recently shown that inhibition of Src kinase results in the accumulation of AQP2 at the plasma membrane, and this process involves phosphorylation of S269, but not S256 [23]. In mouse cortical collecting duct (mpkCCDcl4) it has been reported that wnt5a increases the apical membrane localization of AQP2 without the activation of the classic vasopressin/cAMP/PKA signaling and is instead mediated mainly by phosphorylation at S269 but not S256 [22]. The kinase involved in S/T269 phosphorylation is not yet identified, however, PKA does not phosphorylate this site in vitro [28]. Since S/T269 in AQP2 lies within a ROCK phosphorylation motif RXS/T [22], we tested whether ROCK could phosphorylate in vitro a peptide reproducing the AQP2 C-terminus. Interestingly, we found that ROCK phosphorylates in vitro the T269, and the phosphorylation was almost completely prevented in the presence of the ROCK inhibitor Y27632 (Figure 3). More importantly, we provide evidence that ROCK is responsible for the high pT269-AQP2 levels in living cells expressing V2R-R137L or R137C since inhibition of ROCK reduced pT269-AQP2 levels both in V2R-R137L and R137C compared to the levels found in V2R-wt expressing cells (Figure 3). The observation that ROCK inhibition did not affect V2R-F229V expressing cells, further confirms that V2R-R137L/C and V2R-F229V activate distinct intracellular pathways to promote AQP2 relocation to the plasma membrane. Specifically, V2R-F229V expression seems to exacerbate the activation of the physiological cAMP/PKA axis associated to the activation of V2R-wt, with a consequent increase in PKA-dependent pS256-AQP2, while the V2R-R137L/C rather acts through different mechanisms promoting pS/T269-AQP2 increase (see proposed model in Figure 7).

Of interest, our study provides the novel evidence that in V2R-wt expressing cells, in contrast with what is observed in V2R-R137L/C, ROCK inhibition results in a significant increase in pT269-AQP2 levels. This result is consistent with the hypothesis that, in V2R-wt the ROCK inhibitor, Y27632, causes actin depolymerization and translocation of AQP2 in the absence of the V2R stimulation, as we have previously demonstrated [16,35,39]; and this is paralleled with an increase in pT269-AQP2, probably important to retain the water channel into the plasma membrane. In contrast, the significantly higher basal pT269-AQP2 levels observed in V2R-R137L/C likely result from persistent activation of distinct signaling associated with these gain-of-function V2R variants. Specifically, we provide here evidence that Gα12/13–Rho–ROCK signaling is activated in the V2R-R137L and this pathway is responsible for the increase in pT269-AQP2 levels and the constitutively higher AQP2 cell surface expression and osmotic water permeability. Our findings imply that the V2R-R137L variant of the V2 receptor activates a hitherto unrecognized compartmentalized signaling pathway acting downstream to GPCRs, intersecting the system controlling AQP2 trafficking.

For decades, it has been generally proposed that a given receptor always interacts with a specific G protein or with multiple G-proteins within one family [40,41]. However, it is now clear that several GPCR can couple with distinct unrelated G-proteins leading to the activation of multiple intracellular effectors. The vasopressin receptor family is unique among all classes of peptide receptors in that its individual members couple to different subsets of G proteins [42]. Nevertheless, the functional interaction of V2R with Gα12/13 has never been described. Here we show that the gain-of-function variant V2R-R137L directly or indirectly activates this pathway.

Vasopressin is a peptide very similar to oxytocin with only two amino acid differences and V2R has high homology to oxytocin receptor. Due to the structural similarity of the ligands and the high sequence homology of the receptors, there is some crossreactivity between them. In vitro and in vivo studies have demonstrated that oxytocin receptor can form heterodimers with V2R [43,44], although the physiological relevance of the association of oxytocin receptor with V2R remains to be determined. Interestingly, in human myometrium, oxytocin activates the Rho-ROCK pathway to phosphorylate the regulatory subunit of MLC, leading to contractions [45,46] and vasopressin can also induce uterine contractions [47]. Therefore, it can be speculated that the gain-of-function variant V2R-R137L locks the receptor in a configuration able to directly or indirectly activate a “non-canonical” Gα12/13–Rho–ROCK signaling leading to AQP2 phosphorylation at S/T269 and constitutive increase in the osmotic water permeability which translates into the pathological outcome of NSIAD. Whether the activation of this pathway may explain other much more frequent conditions characterized by water retention associated to elevated pS/T269-AQP2 levels independently from activation of the classical PKA-dependent phosphorylation of AQP2 at S256 deserve to be determined.

Our data failed to demonstrate the activation of the Gα12/13–Rho–ROCK signaling for the V2R-R137C mutant, which however displays elevated pT269-AQP2 levels and sensitivity to ROCK inhibition. Measurements of the basal ROCK activity in the V2R-R137C mutant display a tendency, though not significant, to be higher than V2R-wt. The reason can be related to real functional differences between the two mutants or it might be a difference related to the cell culture model used in this work.

Anyhow, the insensitivity of V2R-R137L/C mutants to vaptans, differently to V2R-F229V and V2R-I130N variants that are sensitive to vaptans [2,10,12,13,36], suggests that Gα12/13–RhoA–ROCK signaling pathway may be potential therapeutic targets for NSIAD in patients carrying these mutations.

In summary, our data demonstrate for the first time that the gain-of-function mutation of the V2R, R137L causing NSIAD, signals through an alternative PKA-independent pathway that increases AQP2 membrane targeting through ROCK induced phosphorylation at T269 independently of S256 of AQP2. We can speculate that under certain conditions a separate signaling pathway from the V2R can increase membrane targeting of AQP2. The physiological relevance of such versatility in signaling associated with V2R variants requires the existence of critical mechanisms of compartmentalization supporting the concept of the multiplicity of G-protein functional coupling.

## References

[1] Feldman B.J., Rosenthal S.M., Vargas G.A., Fenwick R.G., Huang E.A., Matsuda-Abedini M., Lustig R.H., Mathias R.S., Portale A.A., Miller W.L. (2005). Nephrogenic syndrome of inappropriate antidiuresis. N. Engl. J. Med..

[2] Carpentier E., Greenbaum L.A., Rochdi D., Abrol R., Goddard W.A., Bichet D.G., Bouvier M. (2012). Identification and characterization of an activating F229V substitution in the V2 vasopressin receptor in an infant with NSIAD. J. Am. Soc. Nephrol..

[3] Erdelyi L.S., Mann W.A., Morris-Rosendahl D.J., Gross U., Nagel M., Varnai P., Balla A., Hunyady L. (2015). Mutation in the V2 vasopressin receptor gene, AVPR2, causes nephrogenic syndrome of inappropriate diuresis. Kidney Int..

[4] Scheer A., Costa T., Fanelli F., De Benedetti P.G., Mhaouty-Kodja S., Abuin L., Nenniger-Tosato M., Cotecchia S. (2000). Mutational analysis of the highly conserved arginine within the Glu/Asp-Arg-Tyr motif of the alpha(1b)-adrenergic receptor: Effects on receptor isomerization and activation. Mol. Pharmacol..

[5] Audet M., Bouvier M. (2012). Restructuring G-protein- coupled receptor activation. Cell.

[6] Barak L.S., Oakley R.H., Laporte S.A., Caron M.G. (2001). Constitutive arrestin-mediated desensitization of a human vasopressin receptor mutant associated with nephrogenic diabetes insipidus. Proc. Natl. Acad. Sci. USA.

[7] Kocan M., Pfleger K.D. (2009). Detection of GPCR/beta-arrestin interactions in live cells using bioluminescence resonance energy transfer technology. Methods Mol. Biol..

[8] Rochdi M.D., Vargas G.A., Carpentier E., Oligny-Longpre G., Chen S., Kovoor A., Gitelman S.E., Rosenthal S.M., von Zastrow M., Bouvier M. (2010). Functional characterization of vasopressin type 2 receptor substitutions (R137H/C/L) leading to nephrogenic diabetes insipidus and nephrogenic syndrome of inappropriate antidiuresis: Implications for treatments. Mol. Pharmacol..

[9] Takahashi K., Makita N., Manaka K., Hisano M., Akioka Y., Miura K., Takubo N., Iida A., Ueda N., Hashimoto M. (2012). V2 vasopressin receptor (V2R) mutations in partial nephrogenic diabetes insipidus highlight protean agonism of V2R antagonists. J. Biol. Chem..

[10] Tiulpakov A., White C.W., Abhayawardana R.S., See H.B., Chan A.S., Seeber R.M., Heng J.I., Dedov I., Pavlos N.J., Pfleger K.D. (2016). Mutations of Vasopressin Receptor 2 Including Novel L312S Have Differential Effects on Trafficking. Mol. Endocrinol..

[11] Miyado M., Fukami M., Takada S., Terao M., Nakabayashi K., Hata K., Matsubara Y., Tanaka Y., Sasaki G., Nagasaki K. (2019). Germline-Derived Gain-of-Function Variants of Gsalpha-Coding GNAS Gene Identified in Nephrogenic Syndrome of Inappropriate Antidiuresis. J. Am. Soc. Nephrol..

[12] Decaux G., Vandergheynst F., Bouko Y., Parma J., Vassart G., Vilain C. (2007). Nephrogenic syndrome of inappropriate antidiuresis in adults: High phenotypic variability in men and women from a large pedigree. J. Am. Soc. Nephrol..

[13] Ranieri M., Tamma G., Pellegrino T., Vezzi V., Ambrosio C., Gro C., Di Mise A., Costa T., Valenti G., Cotecchia S. (2019). Gain-of-function mutations of the V2 vasopressin receptor in nephrogenic syndrome of inappropriate antidiuresis (NSIAD): A cell-based assay to assess constitutive water reabsorption. Pflug. Arch..

[14] Ranieri M., Di Mise A., Tamma G., Valenti G. (2019). Vasopressin-aquaporin-2 pathway: Recent advances in understanding water balance disorders. F1000Res.

[15] Valenti G.T., Tamma G., Soveral G.N.S., Casini A. (2016). Vasopressin and the Regulation of Aquaporin-2 in Health and Disease. Aquaporins in Health and Disease New Molecular Targets for Drug Discovery.

[16] Nedvetsky P.I., Tamma G., Beulshausen S., Valenti G., Rosenthal W., Klussmann E. (2009). Regulation of aquaporin-2 trafficking. Handb. Exp. Pharmacol..

[17] Jung H.J., Kwon T.H. (2016). Molecular mechanisms regulating aquaporin-2 in kidney collecting duct. Am. J. Physiol. Renal Physiol..

[18] Wilson J.L., Miranda C.A., Knepper M.A. (2013). Vasopressin and the regulation of aquaporin-2. Clin. Exp. Nephrol..

[19] Tamma G., Lasorsa D., Trimpert C., Ranieri M., Di Mise A., Mola M.G., Mastrofrancesco L., Devuyst O., Svelto M., Deen P.M. (2014). A protein kinase A-independent pathway controlling aquaporin 2 trafficking as a possible cause for the syndrome of inappropriate antidiuresis associated with polycystic kidney disease 1 haploinsufficiency. J. Am. Soc. Nephrol..

[20] Olesen E.T., Moeller H.B., Assentoft M., MacAulay N., Fenton R.A. (2016). The vasopressin type 2 receptor and prostaglandin receptors EP2 and EP4 can increase aquaporin-2 plasma membrane targeting through a cAMP-independent pathway. Am. J. Physiol. Renal Physiol..

[21] Olesen E.T., Fenton R.A. (2017). Aquaporin-2 membrane targeting: Still a conundrum. Am. J. Physiol. Renal Physiol..

[22] Ando F., Sohara E., Morimoto T., Yui N., Nomura N., Kikuchi E., Takahashi D., Mori T., Vandewalle A., Rai T. (2016). Wnt5a induces renal AQP2 expression by activating calcineurin signalling pathway. Nat. Commun..

[23] Cheung P.W., Terlouw A., Janssen S.A., Brown D., Bouley R. (2019). Inhibition of non-receptor tyrosine kinase Src induces phosphoserine 256-independent aquaporin-2 membrane accumulation. J. Physiol..

[24] Tamma G., Procino G., Strafino A., Bononi E., Meyer G., Paulmichl M., Formoso V., Svelto M., Valenti G. (2007). Hypotonicity induces aquaporin-2 internalization and cytosol-to-membrane translocation of ICln in renal cells. Endocrinology.

[25] Procino G., Mastrofrancesco L., Mira A., Tamma G., Carmosino M., Emma F., Svelto M., Valenti G. (2008). Aquaporin 2 and apical calcium-sensing receptor: New players in polyuric disorders associated with hypercalciuria. Semin. Nephrol..

[26] Ranieri M., Tamma G., Di Mise A., Russo A., Centrone M., Svelto M., Calamita G., Valenti G. (2015). Negative feedback from CaSR signaling to aquaporin-2 sensitizes vasopressin to extracellular Ca^2+^. J. Cell Sci..

[27] Russo A., Ranieri M., Di Mise A., Dossena S., Pellegrino T., Furia E., Nofziger C., Debellis L., Paulmichl M., Valenti G. (2017). Interleukin-13 increases pendrin abundance to the cell surface in bronchial NCI-H292 cells via Rho/actin signaling. Pflug. Arch..

[28] Hoffert J.D., Fenton R.A., Moeller H.B., Simons B., Tchapyjnikov D., McDill B.W., Yu M.J., Pisitkun T., Chen F., Knepper M.A. (2008). Vasopressin-stimulated increase in phosphorylation at Ser269 potentiates plasma membrane retention of aquaporin-2. J. Biol. Chem..

[29] Moeller H.B., Knepper M.A., Fenton R.A. (2009). Serine 269 phosphorylated aquaporin-2 is targeted to the apical membrane of collecting duct principal cells. Kidney Int..

[30] Procino G., Mastrofrancesco L., Tamma G., Lasorsa D.R., Ranieri M., Stringini G., Emma F., Svelto M., Valenti G. (2012). Calcium-sensing receptor and aquaporin 2 interplay in hypercalciuria-associated renal concentrating defect in humans. An in vivo and in vitro study. PLoS ONE.

[31] He Z., Yang Y., Wen Z., Chen C., Xu X., Zhu Y., Wang Y., Wang D.W. (2017). CYP2J2 metabolites, epoxyeicosatrienoic acids, attenuate Ang II-induced cardiac fibrotic response by targeting Galpha12/13. J. Lipid Res..

[32] Xie S., Mason F.M., Martin A.C. (2016). Loss of Galpha12/13 exacerbates apical area dependence of actomyosin contractility. Mol. Biol. Cell.

[33] Tamma G., Carmosino M., Svelto M., Valenti G. (2005). Bradykinin signaling counteracts cAMP-elicited aquaporin 2 translocation in renal cells. J. Am. Soc. Nephrol..

[34] Vaiskunaite R., Adarichev V., Furthmayr H., Kozasa T., Gudkov A., Voyno-Yasenetskaya T.A. (2000). Conformational activation of radixin by G13 protein alpha subunit. J. Biol. Chem..

[35] Tamma G., Klussmann E., Maric K., Aktories K., Svelto M., Rosenthal W., Valenti G. (2001). Rho inhibits cAMP-induced translocation of aquaporin-2 into the apical membrane of renal cells. Am. J. Physiol. Renal Physiol..

[36] Powlson A.S., Challis B.G., Halsall D.J., Schoenmakers E., Gurnell M. (2016). Nephrogenic syndrome of inappropriate antidiuresis secondary to an activating mutation in the arginine vasopressin receptor AVPR2. Clin. Endocrinol. (Oxf.).

[37] Hoffert J.D., Pisitkun T., Wang G., Shen R.F., Knepper M.A. (2006). Quantitative phosphoproteomics of vasopressin-sensitive renal cells: Regulation of aquaporin-2 phosphorylation at two sites. Proc. Natl. Acad. Sci. USA.

[38] Moeller H.B., Praetorius J., Rützler M.R., Fenton R.A. (2010). Phosphorylation of aquaporin-2 regulates its endocytosis and protein-protein interactions. Proc. Natl. Acad. Sci. USA.

[39] Klussmann E., Tamma G., Lorenz D., Wiesner B., Maric K., Hofmann F., Aktories K., Valenti G., Rosenthal W. (2001). An inhibitory role of Rho in the vasopressin-mediated translocation of aquaporin-2 into cell membranes of renal principal cells. J. Biol. Chem..

[40] Bankir L., Bouby N., Ritz E. (2013). Vasopressin: A novel target for the prevention and retardation of kidney disease?. Nat. Rev. Nephrol..

[41] Natochin Y.V., Golosova D.V. (2020). Vasopressin receptor subtypes and renal sodium transport. Vitam. Horm..

[42] Liu J., Wess J. (1996). Different single receptor domains determine the distinct G protein coupling profiles of members of the vasopressin receptor family. J. Biol. Chem..

[43] Devost D., Zingg H.H. (2004). Homo- and hetero-dimeric complex formations of the human oxytocin receptor. J. Neuroendocrinol..

[44] Muratspahic E., Monjon E., Duerrauer L., Rogers S.M., Cullen D.A., Vanden Broeck J., Gruber C.W. (2020). Oxytocin/vasopressin-like neuropeptide signaling in insects. Vitam. Horm..

[45] Shmygol A., Gullam J., Blanks A., Thornton S. (2006). Multiple mechanisms involved in oxytocin-induced modulation of myometrial contractility. Acta Pharmacol. Sin..

[46] Moore F., Da Silva C., Wilde J.I., Smarason A., Watson S.P., Lopez Bernal A. (2000). Up-regulation of p21- and RhoA-activated protein kinases in human pregnant myometrium. Biochem. Biophys. Res. Commun..

[47] Maggi M., Del Carlo P., Fantoni G., Giannini S., Torrisi C., Casparis D., Massi G., Serio M. (1990). Human myometrium during pregnancy contains and responds to V1 vasopressin receptors as well as oxytocin receptors. J. Clin. Endocrinol. Metab..

